# Effects of dietary formic acid polymer supplementation on growth performance, blood parameters, and intestinal health in lipopolysaccharide-challenged broilers

**DOI:** 10.3389/fmicb.2025.1587832

**Published:** 2025-05-21

**Authors:** Guohui Zhou, Yilin Ge, Yuemeng Fu, Changfei An, Changjin Li, Yang Li, Weiren Yang, Ning Jiao, Jiali Chen

**Affiliations:** ^1^Key Laboratory of Efficient Utilization of Non-grain Feed Resources (Co-construction by Ministry and Province), Ministry of Agriculture and Rural Affairs, Shandong Provincial Key Laboratory of Animal Nutrition and Efficient Feeding, College of Animal Science and Technology, Shandong Agricultural University, Tai’an, China; ^2^SDU-ANU Joint Science College, Shandong University, Wenhuaxilu, Weihai, China; ^3^Bureau of Agriculture and Rural Affairs, Jiangbei Watertown Resort District, Liaocheng, China

**Keywords:** broiler, formic acid, gut microbiota, inflammation, intestinal barrier

## Abstract

This experiment was performed to investigate the impacts of formic acid polymer (FAP) supplementation to the diet on the growth performance, blood metabolites, as well as intestinal barrier function related indicators of broilers under lipopolysaccharide (LPS) stimulation. A total of 450 1-day-old male Arbor Acres broilers with similar body weights were assigned to one of three experimental groups: control (CON) group, basal diet; LPS group, basal diet with LPS (1 mg/kg body weight) challenge; LPS+FAP group, basal diet supplemented with FAP (1,000 mg/kg) and LPS (1 mg/kg body weight) challenge. Each group had 6 replicates of 25 broilers. LPS was injected on days 17, 19, and 21. Samples were collected on day 21, 3 h post-challenge. The experiment lasted 21 days. LPS treatment reduced growth performance, immune function, and caused systemic inflammation, intestinal barrier damage, and microbiota dysbiosis in broilers. However, FAP supplementation significantly reversed these effects by reducing the feed-to-gain ratio and serum levels of interleukin (IL)-1β and tumor necrosis factor-α (*P* < 0.05), while increasing serum levels of complement C4, IL-10, and immunoglobulin M (*P* < 0.05). FAP also improved villus height, trefoil factor family, and mucin 2 levels, decreased caspase activities (*P* < 0.05), and reduced harmful bacteria while promoting beneficial bacteria. To sum up, supplementing 1,000 mg/kg of FAP to the diet effectively enhanced immune function, and mitigated the systemic inflammatory response and intestinal barrier damage caused by LPS, thereby improving broiler growth performance.

## 1 Introduction

A well-functioning gut are not only essential for the digesting and absorbing nutrients, but also serve as a critical defense against pathogen invasion. Pathogens and endotoxins will enter the body through the impaired intestinal barrier, resulting out a systemic inflammatory response and growth retardation (Ghosh et al., 2020). While modern intensive farming offers significant economic benefits, it also makes broilers more vulnerable to harmful bacterial infections, which often lead to intestinal injury (Caekebeke et al., 2020). With the implementation of antibiotic ban policies, it is more urgent to seek novel feed additive to alleviate intestinal barrier inflammatory damage, improving growth performance of broilers.

Over the past few years, organic acids have been commonly used as acidifiers in the livestock industry to improve the production performance of poultry and livestock (Long et al., 2018; Ndelekwute et al., 2021). According to studies, adding organic acids to the diet can enhance broiler chickens’ growth performance and reduce intestinal inflammatory reaction through the TLR4/NF-κB signaling pathway (Dai et al., 2022). Formic acid (FA), as an organic acid, has been widely used in poultry production. It could lower intestinal pH by directly acting on the cell wall of gram-negative bacteria, thus enhancing protein hydrolase activity and improving nutrient digestibility (Dittoe et al., 2018). Moreover, FA could stimulate pancreatic secretion, enhance the activity of digestive enzymes, maintain the stability of the microbial community, selectively inhibit the growth of pathogenic bacteria, and promote the proliferation of beneficial bacteria (Papatisiros et al., 2013). It has been reported that the addition of FA benefits production performance, immune parameters, and intestinal health of broiler chickens (Ragaa and Korany, 2016). However, the application of FA has been limited because of its strong odor and high corrosiveness. Therefore, FA derivatives have gradually received more attention as substitutes for FA due to their non-corrosive and non-irritating characteristics (Ragaa and Korany, 2016; Chen et al., 2017). Two FA molecules are polymerized to form FA polymer (FAP). As a new FA derivative, FAP has the advantages of being non-corrosive, non-irritating, highly stable, and palatable. Previous study showed that dietary addition of 1,000 mg/kg FAP could promote the development of small intestine through suppressing inflammatory responses and altering the gut microbiota composition, then elevating the growth performance of broilers (Li et al., 2022). Zhong et al. (2023) also showed that FAP supplementation elevated the feed conversion ratio and promoted the proliferation of healthy gut microbiota. However, research on FAP is still relatively limited about its alleviating effects on intestinal injury in broiler production.

Lipopolysaccharide (LPS) serves as a principal element of the outer membrane in Gram-negative bacteria, is generally recognized as an activating agent in activating immune responses and is commonly used to establish immune stress model (Chen Y. et al., 2018; Han et al., 2020; Liu H. et al., 2024). LPS administration not only leads to intestinal injury but also has been found to reduce growth performance in broilers (Zheng et al., 2016; He et al., 2022). Previous study in piglets have showed that supplementation of a mixture of FA, benzoic acid, and essential oils reversed LPS-induced intestinal injury and enhanced intestinal barrier integrity via inhibiting inflammation and enhancing antioxidant capacity (Wang et al., 2024c). Based on it, the present study hypothesized that dietary FAP addition also had a protective effect against LPS-induced intestinal damage. Therefore, an LPS-induced intestinal injury model was established to investigate the alleviating effects of adding FAP to the diet on growth performance, blood parameters, and intestinal injury in broiler chickens in this study.

## 2 Materials and methods

### 2.1 Animals, experimental design and diets

A total of 450 1-day-old (day 0 of the experiment) Arbor Acres (AA) male broilers were enrolled in this study, and the average initial body weight (BW) of broilers was 48.47 ± 0.46 g. All the broilers were allocated into three treatment groups at random: CON (basal diet, saline injection), LPS (basal diet, LPS-challenged), and LPS+FAP (basal diet with the addition of 1,000 mg/kg FAP, LPS-challenged). Each group was made up of 6 replicates, and each replicate contained 25 broilers. All broilers were housed in three-tier battery cages (120 × 70 × 40 cm) equipped with dropping trays, trough feeders, and nipple drinkers. Throughout the trial, the broilers were provided with unrestricted access to food and water. The broilers were under continuous lighting conditions for the first 3 days, then until the end of the experiment with 23 h of light and 1 h of darkness. During the first week, the indoor temperature was maintained at 35°C, followed by a gradual decrease of 0.5°C per day. The relative humidity was initially maintained at approximately 60%, and then adjusted to about 50% for the remainder of the trial period. The supplementation level of FAP (Numega Group, Singapore) in the diet of broilers referred to our previous researches (Li et al., 2022; Wang et al., 2022). Basal diets were formulated according to the National Research Council (NRC, 1994) (Table 1). On the morning of days 17, 19, and 21 of the trial (before feeding), all the broilers in the LPS and LPS+FAP groups were given a single intraperitoneal injection of *E. coli* O55:B5 LPS (Sigma-Aldrich, St Louis, MO) at a dose of 1 mg/kg BW, while an equivalent volume of saline solution was administered to the broilers in the CON group, as described by Chen Y. et al. (2018).

**TABLE 1:** Ingredients composition and nutrient levels of basal diets (as-fed basis).

Ingredients, %	1–14 d	15–21
Corn	37.80	40.00
Soybean meal, 46% CP	25.70	23.90
Coarse rice	15.00	12.50
Wheat flour	8.00	8.00
Corn gluten meal	2.00	2.00
Cottonseed meal	4.00	4.00
Hydrolyzed feather meal	1.50	1.50
CaHPO_4_	0.90	0.85
Pulverized Limestone	1.60	1.55
Duck fat	1.50	3.70
Premix^1^	2.00	2.00
Total	100.00	100.00
**Nutrients,%**		
ME, MJ/kg	12.12	12.62
Crude protein	21.62	20.76
Calcium	1.14	1.10
Total phosphorus	0.53	0.51
Lysine	0.87	0.83
Methionine	0.36	0.34
Threonine	0.78	0.75

^1^Provided per kilogram of complete basal diet: 9,600 IU vitamin A; 1,200 IU vitamin D_3_; 24 IU vitamin E; 0.60 mg vitamin K_3_; 2.40 mg vitamin B_1_; 9.60 mg vitamin B_2_; 4 mg vitamin B_6_; 0.01 mg vitamin B_12_; 12.00 mg pantothenic acid; 42.00 mg niacin; 0.21 mg biotin; 0.66 mg folic acid; 1,000 mg choline; 120 mg Mn (MnSO_4_⋅H_2_O); 100 mg Fe (FeSO_4_⋅H_2_O); 100 mg Zn (ZnSO_4_⋅H_2_O); 8.00 mg Cu (CuSO_4_⋅5H_2_O); 0.70 mg I (KIO_3_); 0.30 mg Se (Na_2_SeO_3_).

### 2.2 Preparation and sampling

All the broilers were weighed on the mornings of the 17, 19, and 21 of the experiment, and the feed intake was monitored throughout the feeding trial period and the LPS challenge period. The feed-to-gain ratio was also calculated according to the average daily gain (ADG) and average daily feed intake (ADFI). On the 21st day of the experiment, from each replicate, a chicken with the BW most proximate to the group’s mean was selected. A total of 3 h after LPS challenge, blood samples were taken from the vein in the left wing. The blood was collected into 10 mL procoagulant tube, and then centrifuged at 4,000 × *g* for 10 min at 4°C. The serum was separated and stored at –20°C. Then, the selected broilers were euthanized using carbon dioxide asphyxiation, and a 2-cm section from the middle of the small intestine was collected. The small intestine sample was first rinsed with 0.9% saline and then fixed in 4% paraformaldehyde at room temperature for 24 h. Subsequently, approximately 2 g of small intestinal mucosa were scraped with a sterile glass slide and placed into a 2 mL sterile tube, then immediately frozen in liquid nitrogen for storage. Besides, the cecum contents were collected into a 2 mL sterile tube and stored at –80°C (Li et al., 2022).

### 2.3 Measurement of biochemical parameters in the serum

Serum biochemical indicators, including triglycerides (TG), glucose (GLU), albumin (ALB), total protein (TP), urea nitrogen (UREA), high-density lipoprotein cholesterol (HDL-C), total cholesterol (TCHO), alanine aminotransferase (ALT), and low-density lipoprotein cholesterol (LDL-C). The quantification was carried out using assay kits obtained from Nanjing Jiancheng Bioengineering Institute (Nanjing, China). These measurements were conducted on an automatic chemistry analyzer (Cobas-MiraPlus, Roche Diagnostics, Indianapolis, United States).

### 2.4 Intestinal tissue section analysis

Fixed intestinal segments were first dehydrated using a gradient of ethanol and xylene solutions of varying concentrations, and paraffin embedding was carried out following the standard protocol described by Wright and Manos (1990). Subsequently, sections were cut using a 5 μm microtome blade (Leica Co., Wetzlar, Germany), and the Hematoxylin and Eosin (H&E) staining method was employed for the slides. Finally, villus height (VH) and crypt depth (CD) were measured using an Olympus BX51 microscope (Olympus, Tokyo, Japan) and the JD-801 morphological image analysis system (Jeda Technology Co., Ltd., Nanjing, China), and the VH/CD ratio was calculated (Li et al., 2020b).

### 2.5 Determination of immune and inflammatory indicators levels

The concentrations of serum immunoglobulin (Ig) A, IgM, IgY, and complements C4 and C3 were measured using a commercial ELISA kit (Jiangsu Meimian Industrial Co, Ltd., Jiangsu, China) and determined in terms of the ELISA protocol previously described by Chen et al. (2021a). Chicken ELISA kits (Solarbio Science & Technology Co., Ltd., Beijing, China) were used to examine inflammatory cytokines such as interleukin (IL)-1β, IL-6, IL-10, and tumor necrosis factor (TNF-α) in the serum, in accordance with the manufacturer’s guidelines. Determination of intestinal mucosal barrier integrity and intestinal apoptosis was also performed.

The thawed intestinal tissues were weighed and homogenized using the PBS buffer (1:9, w/v). The supernatants of tissue homogenate were acquired by centrifuging the mixture solution at 5,000 × *g* for 10 min at 4°C, and used to determine the indicators in the intestine. The concentrations of intestinal zonula occludens-1 (ZO-1), trefoil factor family (TFF), mucin 2 (MUC2), and transforming growth factor-α (TGF-α) used for assessment of intestinal mucosal barrier integrity, were assessed via chicken-specific ELISA kits manufactured by Meimian Industrial Co., Ltd. (Yancheng, China) (Wyns et al., 2015). Caspases (caspase-3, -8, and -9) activities in the intestine were detected according to the described method previously by Liu Y. et al. (2024).

### 2.6 Determination of microbial composition and diversity in the cecum

The Omega Bio-tek EZNA TM stool DNA isolation kit (Norcross, Georgia, United States) was employed for the extraction of bacterial DNA from the cecal content. The extracted DNA was evaluated for purity and concentration by 1% agarose-gel electrophoresis (Chen et al., 2021b), after which the primers 515F and 806R were used to amplify the V4 hypervariable region of 16S rDNA, following the method described by Li et al. (2020a). A Qubit 2.0 fluorometer (Thermo Fisher Scientific, United Kingdom) was used to evaluate the quality of the generated library. Library sequencing was conducted on the Illumina HiSeq platform (PE2500, Novogene, Beijing, China), resulting in the generation of 250-bp paired-end reads. The raw paired-end reads were merged with Fast Length Adjustment of Short reads (FLASH, version 1.2.7) (Maran, 2022), and the UCHIME algorithm was employed to eliminate chimeric sequences after comparison with Silva database to acquire the valid sequences (Song et al., 2023). Finally, the effective sequences, which shared over 97% sequence similarity, were grouped into operational taxonomic units (OTUs) using the Uparse software package (Uparse v7.0.1001)^1^ (Edgar, 2013), and subsequently assigned to various taxonomic levels. Taxonomic annotation was carried out through comparison with the Silva database utilizing the Mothur algorithm (Quast et al., 2013). The α and β diversity were analyzed to determine the species richness and diversity, respectively. The principal coordinate analysis (PCoA) profile was employed to visualize the differences in the OTUs matrix based on the Bray-Curtis distance, while significant variations among microbial communities were assessed using an analysis of similarity (ANOSIM).

### 2.7 Statistical analyses

For growth performance analysis, the replicate was regarded as the experimental unit, while the individual broiler was used for other data analyses. The GLIMIX procedure in SAS software (version 9.4; SAS Institute, Cary, NC) was used to analyze the relative abundances of cecal microbiota data, while QIIME 2 and R software (V4.4) were used for alpha and beta diversity data processing and visualization respectively. The other data was analyzed using one-way ANOVA in SAS, with the least significant difference (LSD) method applied for multiple comparisons. Mean ± SEM is used to present the results. A *P*-value < 0.05 indicates a significant difference, while 0.05 ≤ *P* < 0.10 suggests a trend toward significance.

## 3 Results

### 3.1 Effects of dietary FAP addition on the growth performance of LPS-challenged broilers

Table 2 shows that no significant differences were found in ADFI, ADG, and feed-to-gain ratio from d 0 to 17 (*P* > 0.05). Throughout the LPS challenge period (from d 17 to 21), compared with the CON group, the LPS group exhibited a significant reduction in ADG (*P* < 0.05) and a significant elevation in the feed-to-gain ratio (*P* < 0.05). No significant differences were observed between the CON and LPS+FAP groups in terms of ADFI, ADG, and feed-to-gain ratio (*P* > 0.05).

**TABLE 2:** Effects of dietary formic acid polymers (FAP) supplementation on the growth performance of broilers challenged with lipopolysaccharide (LPS).

Items	Treatment			*P*-value
	**CON**	**LPS**	**LPS+FAP**	
**d 0-17**				
ADFI, g	52.28 ± 0.96	55.35 ± 1.45	53.92 ± 0.88	0.323
ADG, g	28.91 ± 2.30	33.24 ± 1.63	30.42 ± 1.59	0.271
Feed-to-gain ratio	1.83 ± 0.12	1.67 ± 0.05	1.79 ± 0.08	0.470
**d 17-21**				
ADFI, g	93.69 ± 3.68	85.03 ± 6.96	84.60 ± 5.39	0.441
ADG, g	63.55 ± 3.70^a^	45.53 ± 3.45^b^	54.77 ± 2.32^ab^	0.005
Feed-to-gain ratio	1.49 ± 0.08^b^	1.86 ± 0.03^a^	1.54 ± 0.04^b^	<0.001

CON (basal diet, saline injection); LPS (basal diet, LPS-challenged); LPS+FAP (basal diet with the addition of 1,000 mg/kg formic acid polymers, LPS-challenged). BW, body weight; ADFI, average daily feed intake; ADG, average daily gain. Data are presented as mean ± standard error (*n* = 6).

^a,b^Different superscripts indicate a significant difference (*P* < 0.05).

### 3.2 Effects of dietary FAP addition on serum metabolites of LPS-challenged broilers

**FIGURE 1:**
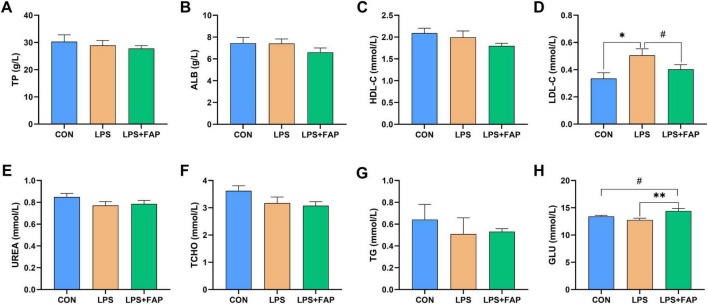
Effects of dietary formic acid polymer (FAP) supplementation on plasma metabolites in broilers challenged with lipopolysaccharide (LPS). **(A)** TP, total protein; **(B)** ALB, albumin; **(C)** HDL-C, high-density lipoprotein cholesterol; **(D)** LDL-C, low-density lipoprotein cholesterol; **(E)** UREA, urea nitrogen; **(F)** TCHO, total cholesterol; **(G)** TG, triglycerides; **(H)** GLU, glucose. CON (basal diet, saline injection); LPS (basal diet, LPS-challenged); LPS+FAP (basal diet with the addition of 1,000 mg/kg FAP, LPS-challenged). A *P*-value < 0.05 was interpreted as indicating statistical significance. *P < 0.05, **P < 0.01, ^#^0.05 ≤ *P* < 0.1. *n* = 6 for each treatment.

In comparison to the CON group, LPS injection markedly elevated serum concentration of LDL-C (Figure 1D) (*P* < 0.05). Under LPS challenge, dietary FAP inclusion significantly increased GLU (Figure 1H) concentration (*P* < 0.05) and exhibited a trend toward reducing LDL-C concentration in the serum (*P* < 0.10). Besides, compared with the CON group, the LPS+FAP group showed a trend of increased serum GLU concentration (*P* < 0.10). No significant differences were observed in TP (Figure 1A), ALB (Figure 1B), HDL-C (Figure 1C), UREA (Figure 1E), TCHO (Figure 1F), and TG (Figure 1G) across the three treatment groups (*P* > 0.05).

### 3.3 Effects of dietary FAP addition on serum supplements, immunoglobulins, and inflammatory cytokines of LPS-challenged broilers

**FIGURE 2:**
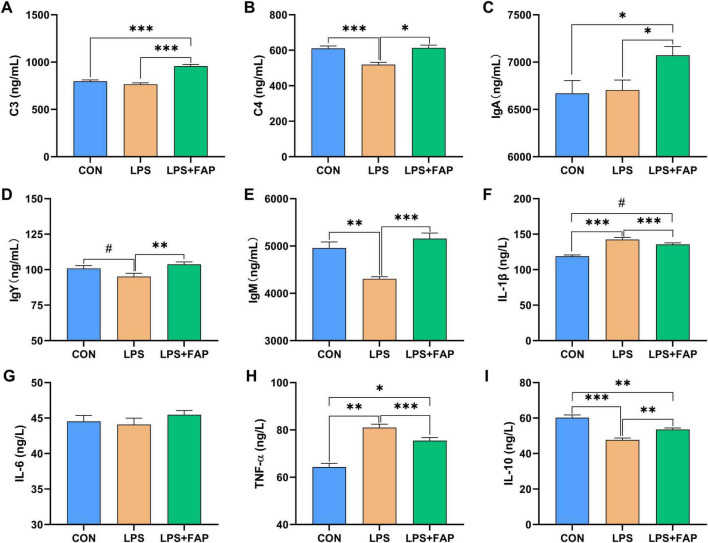
Effects of dietary formic acid polymer (FAP) supplementation on plasma supplements, immunoglobulins, and inflammatory cytokines in broilers challenged with lipopolysaccharide (LPS). **(A)** C3, complement C3; **(B)** C4, complement C4; **(C)** IgA, immunoglobulin A; **(D)** IgY, immunoglobulin Y; **(E)** IgM, immunoglobulin M; **(F)** IL-1β, interleukin-1β; **(G)** IL-6, interleukin-6; **(H)** TNF-α, tumor necrosis factor-α; **(I)** IL-10, interleukin-10. CON (basal diet, saline injection); LPS (basal diet, LPS-challenged); LPS+FAP (basal diet with the addition of 1,000 mg/kg FAP, LPS-challenged). A *P*-value < 0.05 was interpreted as indicating statistical significance. *P < 0.05, **P < 0.01, ***P < 0.001, ^#^0.05 ≤ *P* < 0.1. *n* = 6 for each treatment.

LPS injection led to notable declines in serum complement C4 (Figure 2B), IgM (Figure 2E), and IL-10 (Figure 2I) levels (*P* < 0.05), alongside a significant rise in IL-1β (Figure 2F) and TNF-α (Figure 2H) content (*P* < 0.05) relative to the CON group. It also showed a trend toward reducing IgY content (*P* < 0.10). Compared to the LPS group, FAP supplementation in the diet markedly increased serum complement C3 (Figure 2A), complement C4, IgA (Figure 2C), IgY (Figure 2D), IgM, and IL-10 concentrations (*P* < 0.05), and markedly reduced the concentrations of IL-1β and TNF-α in the serum (*P* < 0.05). Moreover, LPS+FAP group displayed higher serum content of complement C3 (*P* < 0.05), IgA *(P* < 0.05), and IL-1β (*P* < 0.10), along with lower serum levels of IL-10 (*P* < 0.05) compared to the CON group. No significant difference was observed in serum IL-6 (Figure 2G) level among the three treatments (*P* > 0.05).

### 3.4 Effects of dietary FAP addition on intestinal morphometry of LPS-challenged broilers

**FIGURE 3:**
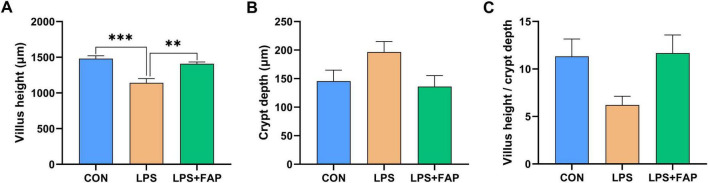
Effects of dietary formic acid polymer (FAP) supplementation on intestinal morphology in broilers challenged with lipopolysaccharide (LPS). **(A)** Villus height; **(B)** Crypt depth; **(C)** Villus height/crypt depth. CON (basal diet, saline injection); LPS (basal diet, LPS-challenged); LPS+FAP (basal diet with the addition of 1,000 mg/kg FAP, LPS-challenged). A *P*-value < 0.05 was interpreted as indicating statistical significance. **P < 0.01, ***P < 0.001. *n* = 6 for each treatment.

Figure 3A shows that dietary FAP supplementation markedly mitigated the decrease in intestinal VH caused by LPS challenge to the level found in the CON group (*P* < 0.05). No statistically significant variations in intestinal CD (Figure 3B) and VH/CD (Figure 3C) across the three treatment groups (*P* > 0.05).

### 3.5 Effects of dietary FAP addition on intestinal barrier function and apoptosis of LPS-challenged broilers

**FIGURE 4:**
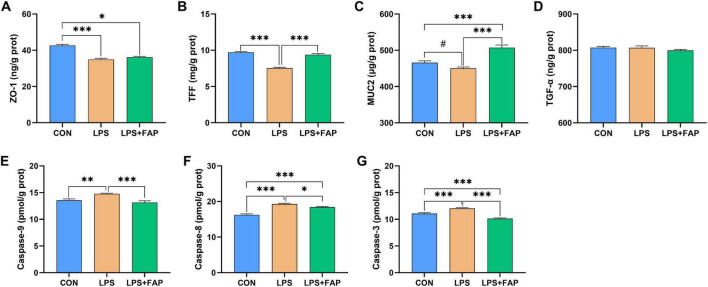
Effects of dietary formic acid polymer (FAP) supplementation on intestinal barrier function and apoptosis in broilers challenged with lipopolysaccharide (LPS). **(A)** ZO-1, zonula occludens-1; **(B)** TFF, trefoil factor family; **(C)** MUC2, mucin 2; **(D)** TGF-α, transforming growth factor-α; **(E)** Caspase-9; **(F)** Caspase-8; **(G)** Caspase-3. CON (basal diet, saline injection); LPS (basal diet, LPS-challenged); LPS+FAP (basal diet with the addition of 1,000 mg/kg FAP, LPS-challenged). A *P*-value < 0.05 was interpreted as indicating statistical significance *P < 0.05, **P < 0.01, ***P < 0.001, ^#^0.05 ≤ *P* < 0.10. *n* = 6 for each treatment.

LPS administration markedly decreased ZO-1 (Figure 4A) and TFF (Figure 4B) concentrations (*P* < 0.05) and showed a trend toward decreasing the MUC2 (Figure 4C) level (*P* < 0.10) in the intestinal mucosa relative with the CON group. However, the addition of dietary FAP significantly elevated the levels of intestinal TFF and MUC2 in broilers subjected to LPS injection (*P* < 0.05). The LPS+FAP group indicated markedly higher MUC2 content in the intestinal mucosa (*P* < 0.05), as well as markedly lower intestinal mucosal ZO-1 level (*P* < 0.05) relative to the CON group. No significant difference was found in the intestinal concentration of TGF-α (Figure 4D) across the three groups (*P* > 0.05). Besides, LPS significantly elevated the activities of caspase-9 (Figure 4E), caspase-8 (Figure 4F), and caspase-3 (Figure 4G) in intestinal mucosa compared with the other two groups (*P* < 0.05). In the LPS+FAP group, caspase-8 activity was significantly elevated (*P* < 0.05), but caspase-3 activity was significantly decreased (*P* < 0.05) compared to those in the CON group.

### 3.6 Effects of dietary FAP addition on cecal microbiota diversity of LPS-challenged broilers

**FIGURE 5:**
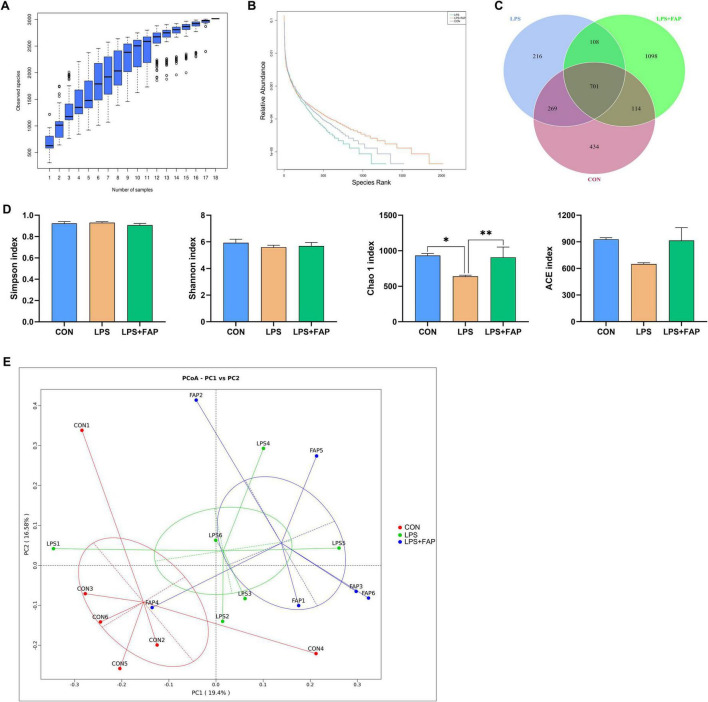
Effects of dietary formic acid polymer (FAP) supplementation on cecal microbiota richness and diversity in broilers challenged with lipopolysaccharide (LPS). **(A)** Rarefaction curve tending to approach the asymptote indicated the sequence depth met the requirements for sequencing and analysis. **(B)** Rank abundance curve reflected the richness of bacterial community by the width of the curve in the horizontal direction. **(C)** Stem-and-leaf display was generated to depict shared and unique sequences among the treatments. **(D)** α diversity indexes, including Shannon, Simpson, Chao 1, and ACE indexes, were used to estimate bacterial community richness and diversity, and values are mean 6 standard error (*N* = 6). **(E)** The principal coordinate analysis (PCoA) profile of weighted Unifrac distance. CON (basal diet, saline injection); LPS (basal diet, LPS-challenged); LPS+FAP (basal diet with the addition of 1,000 mg/kg FAP, LPS-challenged). A *P*-value < 0.05 was interpreted as indicating statistical significance. *P < 0.05, **P < 0.01, # 0.05 ≤ *P* < 0.10. *n* = 6 for each treatment.

The species accumulation curves (Figure 5A) began to plateau when the analyzed samples number reached 18, indicating that the sample size was sufficient for OTU analysis and could reliably estimate species richness. The rank abundance curve (Figure 5B) manifested that the bacterial community richness was highest in the LPS+FAP group, following the order of LPS+FAP > CON > LPS. Similarly, the Venn diagram revealed that there was a total of 701 shared OTUs among the three groups, and the CON, LPS, and LPS+FAP groups had 434, 216, and 1,098 unique OTUs, respectively (Figure 5C). In this study, the cecal microbial α diversity was evaluated with the Simpson, Shannon, Chao 1, and ACE indices (Figure 5D). The Chao 1 indices was markedly reduced in the LPS group compared to the other two groups (*P* < 0.05), and the Chao 1 index did not show a significant difference between the CON and LPS+FAP groups (*P* > 0.05). The PCoA analysis (Figure 5E) indicated that the LPS+FAP group had a distinct separation from the CON and LPS groups. Consistently, the ANOSIM results (Table 3) indicated that the microbial community composition differed significantly between the LPS+FAP group and the CON group (*P* < 0.05).

**TABLE 3:** Analysis of ANOSIM to access beta diversity of broiler cecal microbiota.

Group-pair	*R*-value	*P*-value
CON vs LPS	0.144	0.162
CON vs LPS+FAP	0.276	0.047
LPS vs LPS+FAP	0.016	0.393

CON (basal diet, saline injection); LPS (basal diet, LPS-challenged); LPS+FAP (basal diet with the addition of 1,000 mg/kg formic acid polymers, LPS-challenged). A *P*-value < 0.05 was interpreted as indicating statistical significance. *n* = 6 for each treatment.

### 3.7 Effects of dietary FAP addition on cecal microbiota relative abundance of LPS-challenged broilers

**FIGURE 6:**
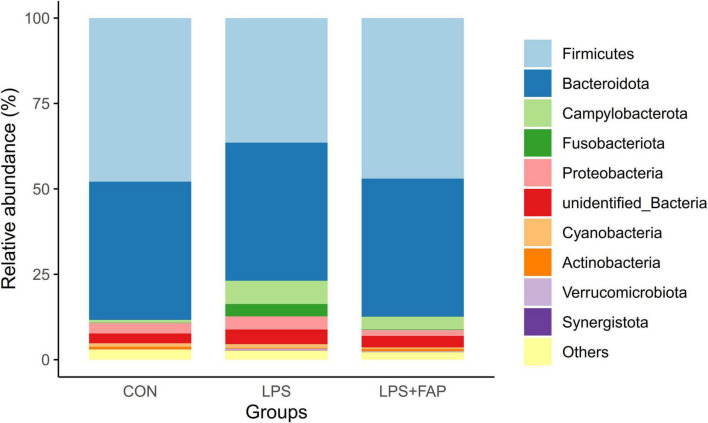
Effects of dietary formic acid polymer (FAP) supplementation on the relative abundance of cecal microbiota at the phylum level in broilers challenged with lipopolysaccharide (LPS). CON (basal diet, saline injection); LPS (basal diet, LPS-challenged); LPS+FAP (basal diet with the addition of 1,000 mg/kg FAP, LPS-challenged). *n* = 6 for each treatment.

**FIGURE 7:**
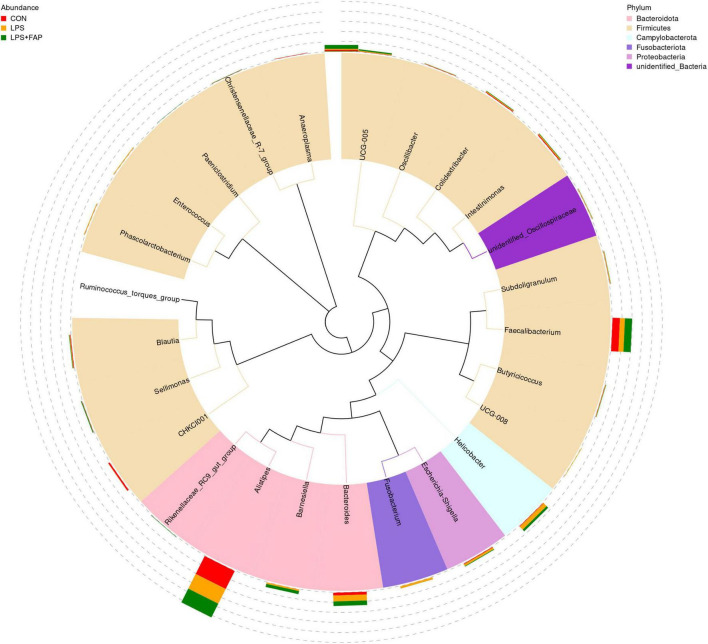
Effects of dietary formic acid polymer (FAP) supplementation on the relative abundance of cecal microbiota at the genus level in broilers challenged with lipopolysaccharide (LPS). The phylogenetic tree created using the sequences from the top 25 genera. The inner circle color-coded branches represent the respective phylum, while the outer circle features a stacked bar chart displaying the relative abundance of each genus across different treatments. CON (basal diet, saline injection); LPS (basal diet, LPS-challenged); LPS+FAP (basal diet with the addition of 1,000 mg/kg FAP, LPS-challenged). *n* = 6 for each treatment.

The changes of cecal microbial relative abundance at the phylum level (top 10) were shown in Figure 6. Firmicutes and Bacteroidetes were the dominant phyla in the CON group and the LPS+FAP group, while Bacteroidetes, Proteobacteria, and Firmicutes were the predominant phyla in the LPS group. Among the top 10 phyla, significant differences were noted in the Campylobacterota, Fusobacteriota, Verrucomicrobiota, and Synergistota relative abundances (Table 4). The LPS challenge caused a marked elevation in the Campylobacterota, Fusobacteriota, Verrucomicrobiota, and Synergistota abundances (*P* < 0.05) relative to the CON group, but dietary FAP addition markedly alleviated LPS-stimulated increases in the relative abundances of Campylobacterota, Fusobacteriota, and Synergistota, bringing them to levels similar to those in the CON group (*P* < 0.05). Figure 7 illustrates the changes in cecal microbial relative abundance at the genus level (top 25), clearly displaying that the dominate genera in the CON group were *Alistipes* and *Faecalibacterium*, in the LPS group were *Alistipes*, *Faecalibacterium*, *Bacteroides*, and *Helicobacter*, while in the LPS+FAP group were *Alistipes*, *Faecalibacterium*, *Bacteroides*, and *[Ruminococcus]_torques_group*. As displayed in Table 4, the LPS group exhibited significant increases in the relative abundances of *Helicobacter*, *Barnesiella*, *Fusobacterium*, *Enterococcus*, and *UCG-008* compared with the CON group (*P* < 0.05). Under LPS challenge, dietary FAP inclusion markedly decreased *Fusobacterium*, *Enterococcus* and *UCG-008* relative abundances in the cecum (*P* < 0.05). Moreover, the FAP group showed markedly higher relative abundances of *Rikenellaceae_RC9_gut_group* and *Barnesiella* (*P* < 0.05), and lower relative abundances of *CHKCI001* and *Anaeroplasma* in the cecum of broilers compared to the CON group (*P* < 0.05).

**TABLE 4:** Effects of dietary formic acid polymers (FAP) supplementation on the relative abundances of differential cecal microbiotas at the phylum and genus levels.

Items (%)	Treatment			*P-*value
	**CON**	**LPS**	**LPS+FAP**	
**Phylum level**				
Campylobacterota	0.81 ± 0.26^b^	6.78 ± 2.98^a^	3.70 ± 2.10^ab^	0.025
Fusobacteriota	0.10 ± 0.04^b^	3.65 ± 1.97^a^	0.16 ± 0.08^b^	0.001
Verrucomicrobiota	0.04 ± 0.01^b^	0.37 ± 0.13^a^	0.21 ± 0.03^a^	<0.001
Synergistota	0.02 ± 0.004^b^	0.14 ± 0.14^a^	0.06 ± 0.04^ab^	0.023
**Genus level**				
*Helicobacter*	0.81 ± 0.26^b^	6.77 ± 2.97^a^	3.67 ± 2.10^ab^	0.026
*Barnesiella*	0.16 ± 0.06^b^	2.31 ± 0.81^a^	5.12 ± 2.77^a^	<0.001
*CHKCI001*	2.49 ± 2.06^a^	0.30 ± 0.21^b^	0.06 ± 0.02^b^	0.003
*Fusobacterium*	0.09 ± 0.03^b^	3.28 ± 1.93^a^	0.16 ± 0.08^b^	0.001
*Enterococcus*	0.15 ± 0.02^b^	0.96 ± 0.86^a^	0.07 ± 0.03^b^	0.012
*Rikenellaceae_RC9_gut_group*	0.05 ± 0.02^b^	0.03 ± 0.01^b^	0.59 ± 0.52^a^	0.008
*UCG-008*	0.03 ± 0.002^b^	0.67 ± 0.46^a^	0.09 ± 0.04^b^	<0.001
*Anaeroplasma*	0.61 ± 0.47^a^	0.04 ± 0.01^b^	0.07 ± 0.03^b^	0.003

CON (basal diet, saline injection); LPS (basal diet, LPS-challenged); LPS+FAP (basal diet with the addition of 1,000 mg/kg formic acid polymers, LPS-challenged). Data are presented as mean ± standard error (*n* = 6). Means with different superscripts within a row differ (*P* < 0.05).

## 4 Discussion

Over the past six decades, genetic selection of broilers has primarily focused on production traits like growth rate and feed conversion to improve efficiency and meet market demands (Zuidhof et al., 2014). Besides, modern poultry production further accelerates the growth rates of broilers, coupled with the limited and intensive spaces, making the intestinal barrier more susceptible to bacterial infections (Yuan et al., 2023). Finally, the fast-growing broilers are easily prone to the intestinal damage, leading to the decreased growth performance and even death (Lv et al., 2025). In this study, LPS administration decreased the ADG and increased the feed-to-gain ratio in broilers, aligned with the results of previous studies (Chen Y. et al., 2018; Shi et al., 2022). Dietary FAP supplementation did not improve the growth performance of broilers from day 0 to 17, but suppressed the adverse effects on growth performance induced by LPS challenge from d 17 to 21. Moreover, we also found that the feed-to-gain ratio value during 0-17 d was higher than that during days 17-21 d in the CON and LPS+FAP groups. This result may be attributed to the underdeveloped digestive system of broiler chickens aged 0-17 days, during which most energy is allocated to maintaining body temperature and supporting organ development; in contrast, broilers between 17 and 21 days exhibit more vigorous metabolism, accelerated muscle growth, and enhanced physiological adaptability (Wang et al., 2024b; Cordero et al., 2025). In summary, the results suggested that 1,000 mg/kg FAP supplementation could mitigate LPS challenge-induced decrease in growth performance of broilers in this study.

Serum biochemical parameters are reliable indicators of health status of animals. In this study, LPS challenge elevated serum LDL-C concentration of broilers, in accordance with Chen et al. (2024). High plasma concentrations of LDL-C substantially contribute to cardiovascular risk, making LDL-C lowering an essential preventive measure against atherosclerosis-related diseases (Chen et al., 2023). LPS stimulation can trigger the increase of inflammatory cytokines, such as TNF-α, by activating the TLR4 signaling pathway (Liu C. et al., 2019). These inflammatory cytokines may inhibit the expression of PCSK9 protein, thereby weakening the degradation effect on LDL receptor (Zhang et al., 2016). Consequently, the clearance rate of circulating LDL-C is reduced, leading to the elevated serum LDL-C levels (Xiao et al., 2012; Khanmohammadi and Kuchay, 2022). Consistently, we also observed increased pro-inflammatory cytokines TNF-α and IL-1β, and decreased anti-inflammatory cytokine IL-10, as well as complement C4, IgY, and IgM, in the serum of LPS group. The complement components and immunoglobulins generated by the liver and circulating in the plasma play a vital role in protecting from bacterial infections and providing passive immunity (Hu et al., 2024). Complement C4 was the most polymorphic protein in complement system, and decreased serum level of complement C4 was often found in chronic active hepatitis. IgY is one of the major antibodies in avian serum and the dominant isotype in secondary immune responses (Pacheco et al., 2023). IgM, the primary antibody isotype that appears in evolution, ontogeny, and immune responses, not only performs immune regulation but also plays a key role in immune balance (Liu J. et al., 2019). Wu et al. (2021) also indicated that LPS challenge decreased serum IgY and IgM of broilers. The above results indicated that LPS injection impaired the immune function and resulted in the systemic inflammatory response in broilers. However, we found that the addition of dietary FAP mitigated the LPS-induced declines in the serum C4, IgY, and IgM concentrations, and increased serum C3 and IgA content in broilers in this study. Complement C3 is a crucial component of the innate immune system, which is important for immune defense and provides a link between innate and adaptive immunity (Fang et al., 2023). IgA is the most abundant isotype of immunoglobulin secreted into mucosal tissues (mainly intestinal mucus), and it can bind to intestinal microbial antigens to limit their toxins and thus reduce inflammation (Guo et al., 2021). Collectively, our findings demonstrated that not only did dietary FAP addition alleviate LPS-induced impairment of immune function and systemic inflammatory response, but also further enhanced immune functions of broilers.

The small intestine is both the principal site for nutrient digestion and absorption and serves as a crucial defense barrier for the body. The VH is a key factor in determining the small intestine’s ability to absorb nutrients (Beumer and Clevers, 2021). In the current study, LPS-challenged broilers exhibited a reduction in VH, which is consistent with the earlier work conducted by Chen Y. et al. (2018). Besides, we also found that LPS challenge decreased intestinal ZO-1, TFF, and MUC2 content in broilers, which was in line with the previous studies (Wen et al., 2022; Liu H. et al., 2024). The intestinal barrier, primarily constituted of a monolayer of intestinal epithelial cells and intercellular tight junction proteins, represents a complex structure essential for maintaining immune homeostasis, safeguarding against intestinal infections, and facilitating nutrient absorption (Chen J. et al., 2018). Various stimuli, including pathogenic agents and vaccination, can compromise the integrity of the intestinal barrier, leading to heightened intestinal permeability (Liu H. et al., 2024). The regulation of intestinal permeability is predominantly mediated by tight junction proteins, including occludin, claudins, and ZO-1 (Lin et al., 2025). The TFF consists of a group of small peptides that are usually secreted along with mucins and are highly expressed in the mucous membranes lining the gastrointestinal tract (Hoffmann, 2020). The MUC2, an important secretory protein found in the gut, helps protect the gut barrier, maintain microbiome balance, and prevent diseases (Yao et al., 2021). Studies have shown that up-regulation of MUC2 and TFF levels has a positive effect on improving the intestinal barrier (Wu et al., 2020). There results demonstrated that LPS destructed the intestinal barrier of broilers. Excessive apoptosis of epithelial cells is regarded as a significant factor to the damage of the intestinal mucosal barrier. Cell apoptosis is typically regulated through two principal mechanisms: the intrinsic pathway and the extrinsic pathway (Zhu et al., 2013). The intrinsic pathway, also known as the mitochondrial pathway, is mediated by mitochondria and is specifically characterized by the caspase-9 activation (Djiadeu et al., 2017). In contrast, the extrinsic pathway, which depends on membrane death receptors, is specifically characterized by the caspase-8 activation (Locquet et al., 2021). Both caspase-8 and -9 can subsequently activate caspase-3, leading to apoptosis (Xiao et al., 2025). Collectively, LPS injection could damage intestinal morphology and mucosal barrier of broilers via inducing intestinal apoptosis in this study. Nevertheless, dietary FAP addition mitigated the adverse effects on intestinal morphology and mucosal barrier, and inhibited intestinal activities of caspase-9, -8, and -3 in broilers. Sabour et al. (2019) also found that 1 g/kg OA addition elevated the intestinal VH. Abnormal activation of intestinal cell apoptosis can lead to intestinal cell damage and tissue degradation (Wang et al., 2024a). Previous studies showed that intestinal damage could be alleviated by inhibiting apoptosis (Jiang et al., 2021; Cui et al., 2023). Thus, our results showed that adding dietary FAP could reduce intestinal injury by inhibiting apoptosis in this study.

Intestinal barrier is crucial in blocking the infiltration of harmful bacteria and substances through the intestinal wall. Compromise of intestinal barrier can disrupt the homeostasis of the intestinal microbiota. In this study, broilers exposed to LPS exhibited a decrease in the Chao 1 index, but did not affect the β diversity, in comparison to the CON broilers. Wang et al. (2024d) also indicated that short-term intraperitoneal injection of LPS did not change the β diversity of cecal microbiota. The Chao 1 index is often utilized to estimate community richness. Zhou et al. (2023) also showed that LPS administration decreased Chao 1 index in the ileum of laying hens. Previous studies have reported that a reduction in α diversity is commonly associated with inflammatory enteritis (Li et al., 2022; Wen et al., 2022). Reduced α-diversity is often associated with increased abundance of pathogenic bacteria related to intestinal inflammation (Wei et al., 2022). Consistently, LPS injection increased the abundances of phyla Campylobacterota, Fusobacteriota, and Synergistota and the genera *Helicobacter* and *Fusobacterium*, and decreased the relative abundances of *CHKCI001* and *Anaeroplasma*. It has been proven that increased abundances of Campylobacterota, Fusobacteriota, and *Helicobacter* are associated with ulcerative colitis (Metwaly and Haller, 2024). A prior study in piglets indicated that the increase of *Anaeroplasma* abundance was beneficial to the intestinal health and the improvement of growth performance (Ribeiro et al., 2024). Additionally, the fecal relative abundance of *CHKCI001* was conducive to improving egg production and feed conversion rate (Deng et al., 2022). The above results suggested that LPS injection resulted in the invasion and proliferation of harmful bacteria in this study, which inhibited the proliferation of beneficial bacteria. Nevertheless, dietary FAP addition inhibited the reduction of Chao 1 index of cecal microbiota caused by LPS challenge, and changed the β diversity compared to the CON group. Moreover, dietary FAP supplementation not only suppressed the increases in relative abundances of phyla Campylobacterota, Fusobacteriota, and Synergistota and the genera *Helicobacter* and *Fusobacterium*, but also decreased the abundance of harmful bacteria *Enterococcus* in the LPS-challenged broilers. Abd El-Hack et al. (2024) also found that adding FA to the diet reduced the levels of harmful bacteria, such as *Coliform* and *E. coli*. Previous studies have demonstrated that dietary supplementation with 0.25-0.50% benzoic acid can positively modulate the intestinal microbiota by promoting beneficial bacteria such as Lactobacillus, reducing Escherichia coli populations, and improving intestinal morphology (Choi and Kim, 2024). Some scholars also found that the addition of FA and monolaurin to feed reduced Enterobacteriaceae abundance but increased the number of *Lactobacillus* in weaned pigs challenged with *E. coli* CVCC225 (Ren et al., 2020). Above all, this study suggested that dietary supplementation of FAP could alleviate LPS challenge-induced gut microbiota dysbiosis through the suppression of harmful bacteria proliferation and the promotion of beneficial bacteria growth.

## 5 Conclusion

To sum up, adding 1,000 mg/kg FAP to the diet could mitigate the systemic inflammatory response and gut barrier damage by LPS-induced, thus improving the growth performance of broilers. Therefore, FAP is expected to become an innovative feed additive in the future to maintain gut health and improve production performance.

## Data Availability

The sequencing data are stored in the NCBI Sequence Read Archive (SRA) database under accession PRJNA1164798 (Illumina sequences).

## Footnotes

1
http://www.drive5.com/uparse/
